# ^129^Xe Dynamic Nuclear Polarization Demystified: The Influence of the Glassing Matrix on the Radical Properties

**DOI:** 10.1021/acs.jpclett.4c00177

**Published:** 2024-03-07

**Authors:** Emma Wiström, Jean-Noël Hyacinthe, Thanh Phong Lê, Rolf Gruetter, Andrea Capozzi

**Affiliations:** † LIFMET, Institute of Physics, École Polytechnique Fédérale de Lausanne (EPFL), Station 6, 1015 Lausanne, Switzerland; ‡ HYPERMAG, Department of Health Technology, Technical University of Denmark, Building 349, 2800 Kgs Lyngby, Denmark

## Abstract

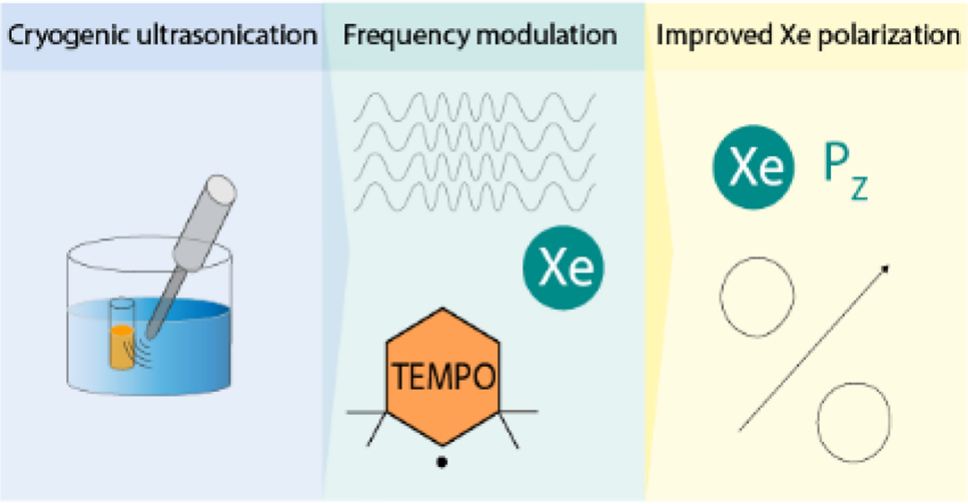

^129^Xe dissolution dynamic nuclear polarization (DNP) is a controversial topic. The gold standard technique for hyperpolarized xenon magnetic resonance imaging (MRI) is spin exchange optical pumping, which received FDA approval in 2022. Nevertheless, the versatility of DNP for enhancing the signal of any NMR active nucleus might provide new perspectives for hyperpolarized ^129^Xe NMR/MRI. Initial publications about ^129^Xe DNP underlined the increased complexity in the sample preparation and lower polarization levels when compared to more conventional ^13^C-labeled molecules, at same experimental conditions, despite very close gyromagnetic ratios. Herein, we introduce, using a Custom Fluid Path system, a user-friendly and very robust sample preparation method. Moreover, investigating the radical properties at real DNP conditions by means of LOngitudinal Detected Electron Spin Resonance, we discovered a dramatic shortening of the electron spin longitudinal relaxation time (*T*_1e_) of nitroxyl radicals in xenon DNP samples’ matrices, with respect to more commonly used water:glycerol ones. Mitigating those challenges through microwave frequency modulation, we achieved over 20% ^129^Xe polarization without employing any deuterated solvent.

The combination of broad chemical shift dispersion, extreme sensitivity to the local environment and relatively long longitudinal relaxation time (*T*_1_) make hyperpolarized (HP) ^129^Xe gas, in combination with magnetic resonance (MR) spectroscopy and imaging, a versatile and powerful analytical tool employed in material science, analytical chemistry, and biomedicine.^1,2^ Without a doubt, investigation of human lung function by means of ventilation imaging is its most widespread application.^3^ To this regard, the Food and Drug Administration (FDA) recently approved Polarean’s XENOVIEW as ionizing radiation-free alternative to ^133^Xe scintigraphy for adults and pediatric patients aged 12 years and older.^4^ Moreover, xenon gas’s great affinity for hydrophobic molecular environments makes it an ideal blood tracer for perfusion MRI.^5^ Notably, it was recently demonstrated that HP ^129^Xe lung perfusion could detect lower gas transfer to the red blood cells in patients with long-COVID, despite having normal CT findings.^6^

Spin exchange optical pumping (SEOP) is the mainstream technique used to hyperpolarize ^129^Xe.^7^ The polarization transfer happens directly in the gas state, inside a so-called “pumping cell” held within a magnetic field of a few millimeters of energy, during collisions between xenon nuclei and Rb atoms previously excited using circularly polarized laser light. If the difficulty in rapidly generating large volumes of ^129^Xe with high polarization has been, for many years, the limiting factor in the widespread application of this technique where a high throughput is required (e.g., clinical MRI), recent technological advancements have circumvented this drawback.^8−11^ To date, one of the most performant available systems can produce up to 300 cm^3^ of ^129^Xe with a nuclear spin polarization (*P*_Xe_) of ∼30% in 5 min (i.e., throughput of 3.6 L/h).^12^

A less common way to generate HP ^129^Xe is dissolution dynamic nuclear polarization (dDNP), followed by a sublimation step to separate the gas from the liquid phase of the dissolved sample.^13^ Different from SEOP, in dDNP, the polarization transfer to the nuclei happens in the solid state at low temperature (0.8–1.4 K) and much higher magnetic fields (3.35–7 T), by means of microwave irradiation.^14^ Unpaired electron spins in the form of organic free radicals represent the source of the polarization. Generally, they are added to the sample by chemical doping.^15,16^ More recently, other strategies involving polarizing matrices^17,18^ and nonpersistent photoinduced radicals^19−24^ were developed with the intent of facilitating radical filtration and enabling transport of hyperpolarization.^25−28^

^129^Xe DNP is a controversial topic. Initially presented as a method with great potential for applications where a high volume of HP xenon gas is needed, because of the more than 500-times expansion coefficient between solid state and gas state,^13^ it later presented a strong limitation in the amount of gas that can be dissolved in the radical-doped hosting glassing solvent (i.e., ethanol, isobutanol, 2-methyl-1-pentanol) representing the “scaffold” of the DNP sample.^29,30^ Moreover, sample preparation is cumbersome and hard to control, increasing the chance of dealing with inhomogeneous samples with poor DNP performance, with respect to more conventional ^13^C ones.^30,31^ Nevertheless, volumes allowing preclinical studies (e.g., 50 mL) could be achieved with an average ^129^Xe polarization of 7%–8%.^13,29^ This value could be improved by reducing the xenon concentration in the solid sample (i.e., a smaller final volume after extraction) and using deuterated solvents, obtaining a gas magnetization high enough to measure HP MRI in a phantom at 9.4 T.^29^ At the same time, DNP is not compound-specific and can enhance the polarization of any NMR active nucleus, hence offering the chance to perform HP ^13^C, ^1^H, ^6^Li, and ^129^Xe MR with one single machine.^29,32−35^ Last but not least, understanding why ^13^C DNP outperforms ^129^Xe at the same experimental conditions and radical concentration, despite the gyromagnetic ratios being very close, can shed some light on the key parameters driving an efficient polarization transfer from the electron to the nuclear spins, providing renewed theoretical insight.

The first aim of this study was to make xenon DNP sample preparation simpler and more robust. We built a setup that, by means of ultrasonication at constant temperature close to the gas’ triple point, can keep the xenon and the radical-doped solvent continuously in the liquid phase during mixing. Moreover, the preparation can happen directly into the sample cup of the DNP system (see the Materials and Methods section for details). This strategy had one immediate consequence: the procedure became less user-dependent, eliminating some tedious manual steps (see Video 1 in the Supporting Information). Most importantly, our hypothesis was that by employing ultrasonication at cryogenic temperature, the homogeneity of the sample would improve.

To quantify that, we investigated the FIDs’ shape of the xenon DNP samples. In Figure 1A, we report the DNP-enhanced time domain acquisition for two samples, one prepared using ultrasonication at controlled temperature of the bath, violet markers, and the other using the traditional procedure, green markers (see the Materials and Methods section for details about the preparation procedures). As one would expect in a solid,^36^ both decays followed a Gaussian behavior nicely. The curve described by the equation *y* = *A* exp(−(*x* – μ)^2^/(2*T*_2_^2^)) could fit the two datasets with a *R*^2^ = 0.999 in both cases, where *A* is a proportional coefficient, μ the center of the Gaussian curve, and *T*_2_ the apparent transversal relaxation time of the sample, related to the half-width at half-maximum (HWHM) of the time course of the signal by the relation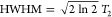 In a solid, the FID’s decay is Gaussian rather than exponential, because different spin populations exist in the sample as a consequence of the anisotropic interactions among the nuclear spins themselves (homonuclear and heteronuclear dipolar interaction) and with the surroundings (chemical shift anisotropy and radicals). The FID (or the spectrum) can be seen as a convolution of exponential relaxation curves (i.e., convolution of Lorentzian curves for the spectrum) with different weights depending on the spin population resonating at a given frequency.^37^ A shorter *T*_2_ value entails a broader distribution of different resonance frequencies. Ultrasonication increased the *T*_2_ by 25% from 36 ± 1 μs to 45 ± 1 μs. Most likely, this effect was due to a more even distribution in the solid matrix of paramagnetic centers and target nuclei. Indeed, at identical radical concentration (i.e., 50 mM), the sample characterized by a shorter *T*_2_ achieved lower polarization with a faster buildup that slightly deviated from a monoexponential behavior (Figure 1B). This could be the symptom of parts of the sample having higher local radical concentration,^23,38^ which speeds up the DNP process, especially at the beginning, but also induces unwanted relaxation of part of the nuclear spin order.

**Figure 1:**
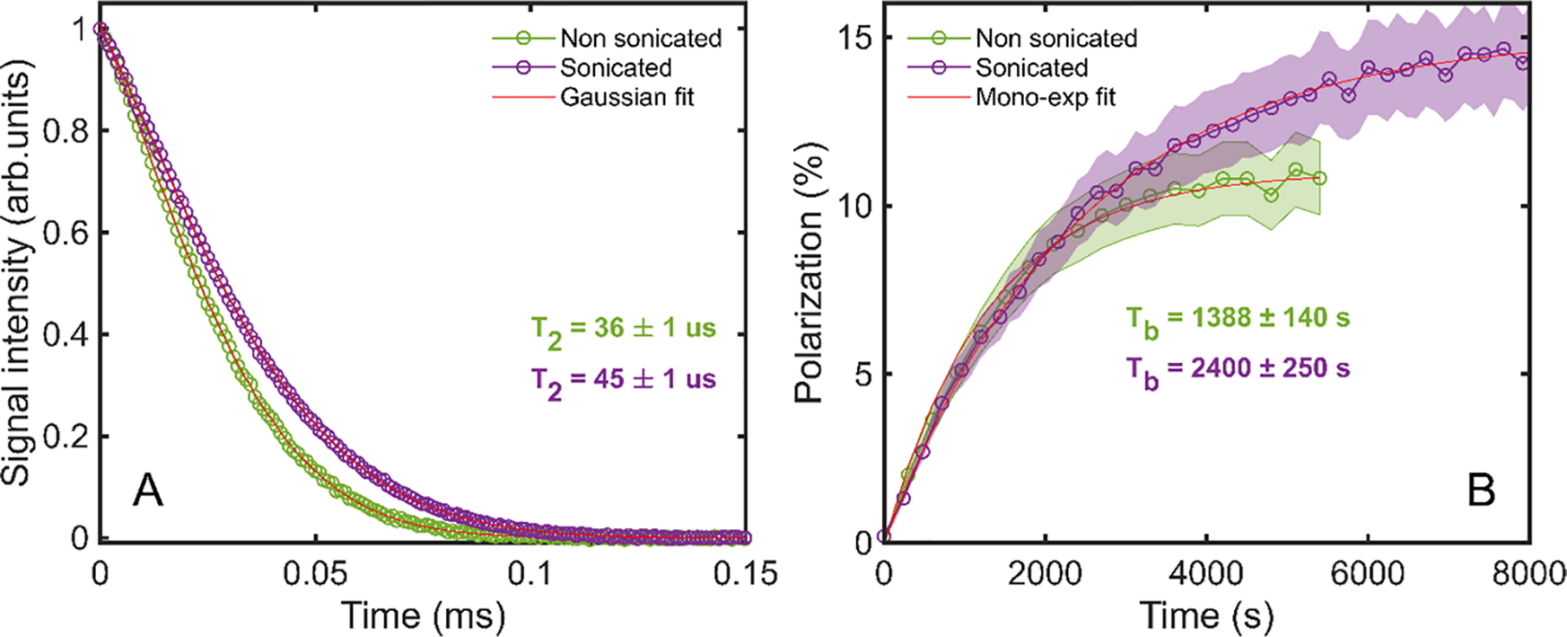
(A) FID comparison and (B) DNP buildup comparison between a ^129^Xe DNP prepared directly inside the CFP’s vial using ultrasonication in melting ethanol (violet markers) and one using the traditional procedure (green markers).^29^ The red curves represent the model used to fit the data; shaded areas represent the error on the data points. The text in bold represents the time constants of the different samples, according to the color code.

As mentioned in the introduction of this Letter, xenon DNP samples usually underperform, with respect to carbon ones under the same experimental conditions and radical concentration. Besides the preparation procedure, the most crucial difference is in the nature of the glassing solvent, or solvent mixture, used to obtain an amorphous phase in the solid state after freezing. Because of the low xenon’s temperature triple point (i.e., 161.38 K) and its hydrophobic nature,^39^ routinely used mixtures such as glycerol:water cannot be employed. Forming an amorphous phase is critical for good DNP, because it promotes spectral diffusion, especially in inhomogeneous broadened spectra. The dipolar interaction couples radical molecules that are close in space, but far in resonance frequency, because of their random orientation, with respect to the direction of the magnetic field.^40^ At the same time, *T*_1e_ strongly affects spectral diffusion:^41^ a spin–lattice relaxation rate faster than the diffusion (or cross-relaxation) rate will not allow spreading of the hole burned by the microwave irradiation across the entire ESR line, leading to poor DNP performance.^40^

In Figure 2A, we report the *T*_1e_ of TEMPO/TEMPOL dissolved in isobutanol/GW55 with or without the presence of the substrate (i.e., xenon for isobutanol, sodium [1-^13^C]acetate for GW55). At the same radical concentration (i.e., 30 mM), the difference in relaxation time between the two solvents was striking. While, in GW55, we measured a *T*_1e_ to be 209 ± 8 ms (blue markers); this value dropped to 40 ± 3 ms in isobutanol (red markers).

**Figure 2:**
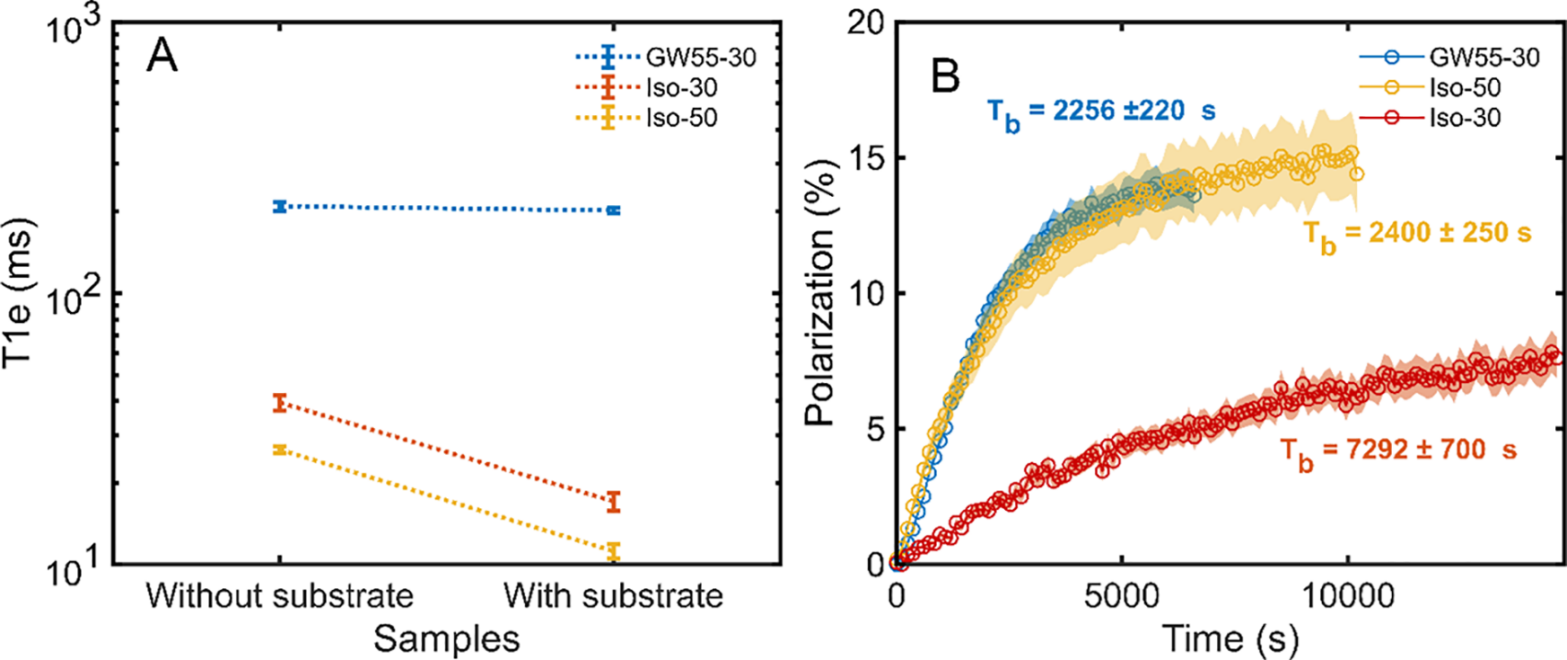
Radical relaxation time as a function of kind of solvent, concentration, and presence of the substrate. The dotted lines help guiding the eye (panel (A)). DNP buildup at optimal monochromatic microwave irradiation for 2.6 M xenon in isobutanol with 30 mM TEMPO, 2.6 M xenon in isobutanol with 50 mM TEMPO, and 3 M sodium [1-^13^C]acetate with 30 mM TEMPOL. The text in bold represents the time constants of the different samples, according to the color code (panel (B)).

Moreover, adding sodium [1-^13^C]acetate to GW55 had essentially no effect (*T*_1e_ = 202 ± 4 ms), while incorporating xenon into the isobutanol sample made the relaxation time value decrease by more than half (*T*_1e_ = 17 ± 1 ms). The latter was in good agreement with the fact that xenon strongly affects the properties of paramagnetic molecules in its surroundings.^45^ This more than 10-fold shorter radical relaxation time had an immediate consequence on the DNP. In Figure 2B, we report the polarization buildup at optimal monochromatic microwave irradiation for these two samples. While the carbon sample could reach 14.2% ± 0.5% after 2 h, the xenon sample barely reached 7.8% ± 0.8% after more than 4 h, suggesting partial saturation and consequent use of only a small part of the available electrons.^46^ Increasing the TEMPO concentration in isobutanol to 50 mM, on the one hand, decreased the *T*_1e_ even further (Figure 2A, yellow markers), while, on the other hand, it increased the dipolar coupling between electron spins, promoting better spectral diffusion. This was reflected on the DNP performance, with the xenon sample doped with 50 mM TEMPO achieving double polarization with respect to the 30 mM TEMPO one and with a buildup time constant of 2400 ± 250 s (Figure 2B, yellow markers).

Although the precise underlying mechanisms for the faster electron spins relaxation in xenon samples are still unclear and would require further investigation, we can certainly consider that, differently from nuclear spins, the most effective relaxation mechanism for electron spins in the solid state is the coupling with lattice vibrations (i.e., phonon modes).^42^ The phonons in a solid, be it a crystal or a glass, are a basic ingredient in understanding such properties as specific heat, melting, ferroelectricity, and superconductivity.^43^ The melting temperature of isobutanol (i.e., 165 K) is much lower compared to the one of glycerol:water at equal volume percentage (i.e., 245 K).^44^ Therefore, the phonon spectral density of the two glassing matrices is likely to be very different, with the intensity of the phonon spectral density around the electron spin resonance at 5 T (i.e., 140 GHz) being stronger for isobutanol than for glycerol:water. Within the same reasoning, further shortening of the *T*_1e_ upon admixture of xenon in the matrix is coherent with the fact that the gas melting point falls at 161 K.

In case of partial saturation of the ESR line and consequent suboptimal DNP enhancement,^46^ working at high microwave power can circumvent this drawback, and it is common practice in MAS-DNP.^47^ Nevertheless, under low-temperature dissolution DNP conditions, increasing the microwave power too much can be counterproductive, generating local heating of the sample and a consequent decrease in the enhancement.^48^ At higher magnetic field (≥6.7 T), where radical *T*_1e_ becomes shorter,^49^ microwave frequency modulation was proved to be useful to increase the solid-state enhancement of ^13^C and ^1^H nuclei.^23,50,51^ With that in mind, we employed microwave frequency modulation on our system at 5 T and 1.20 K to compensate for the prohibitively short relaxation properties of TEMPO in isobutanol. In Figure 3, we report the microwave sweeps, with and without frequency modulation, for 30 mM TEMPO in isobutanol with 2.6 M xenon (Figure 3A), 50 mM TEMPO in isobutanol with 2.6 M xenon (Figure 3B), and 30 mM TEMPOL in GW55 with 3 M sodium [1-^13^C]acetate (Figure 3C). Moreover, for each sample, we overlaid the LOD-ESR spectrum on the DNP sweeps. Looking at the nonmodulated sweeps, the common feature among the samples was that the DNP spectrum nicely followed the ESR one, and the polarization transfer happened at frequencies where also electron spins were resonating. This suggested that the mechanism behind was triple-spin-flips-driven thermal mixing and/or cross-effect.^52,53^

**Figure 3:**
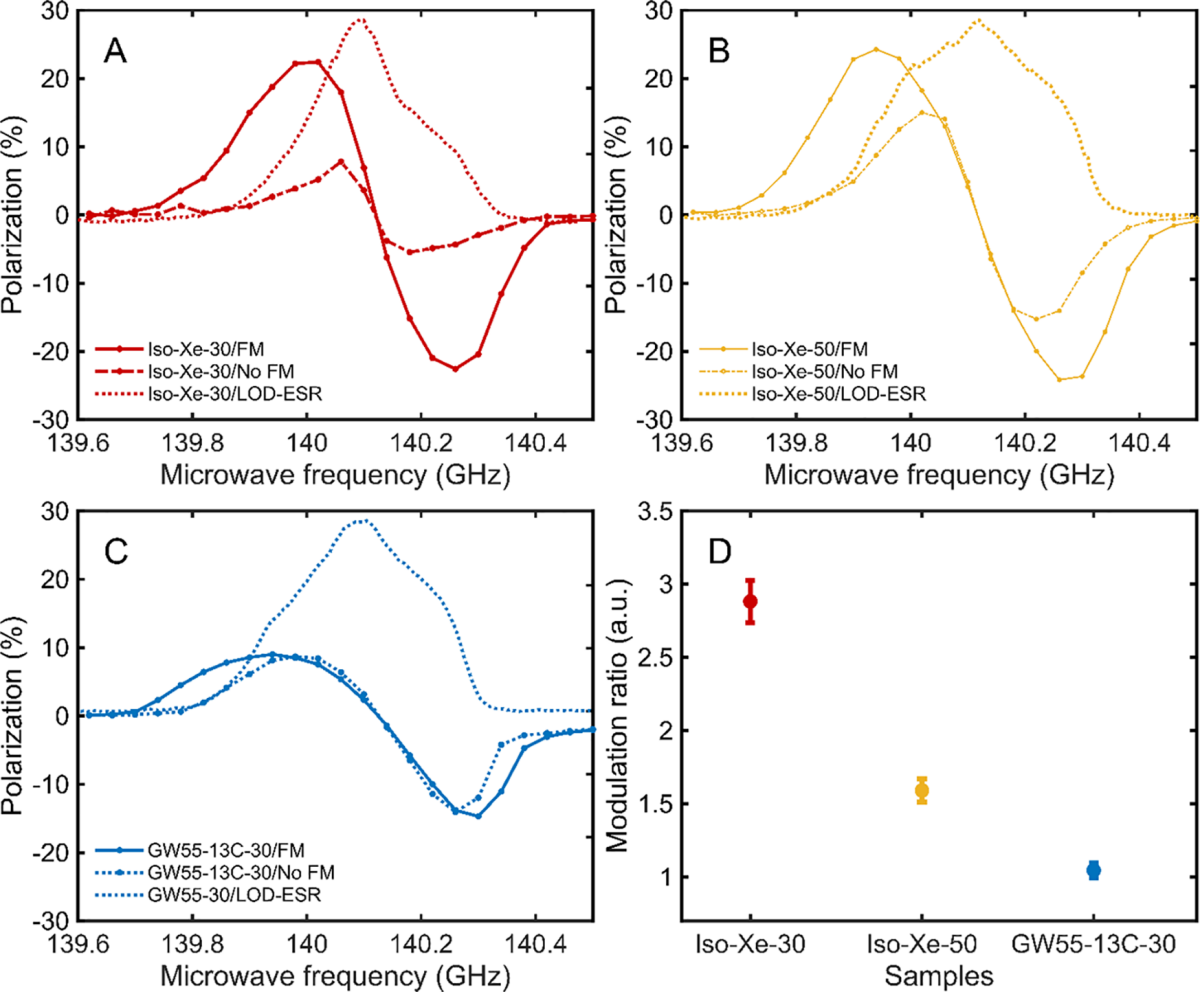
Microwave frequency sweep with and without frequency amplitude modulation (1 kHz rate, 100 MHz amplitude) overlaid to LOD-ESR (arbitrary units) spectrum for (A) 30 mM TEMPO in isobutanol with 2.6 M xenon, (B) 50 mM TEMPO in isobutanol with 2.6 M xenon, and (C) 30 mM TEMPOL in GW55 with 3 M sodium [1-^13^C]acetate. (D) Enhancement’s improvement by applying microwave frequency modulation for the three samples. Because of the short polarization time (see the Materials and Methods section) at each frequency, the polarization values reported on the DNP sweeps (*n* = 1) were adjusted according to the plateau values of full buildups for the different samples (see the Supporting Information).

However, the effects of microwave frequency modulation were very different. While, in Figure 3C, the ^13^C-labeled sample did not show any real improvement, with the maximum achievable polarization remaining at ∼14.5% and only moving toward lower (higher) frequency for the positive (negative) maximum; this is an important effect that was observed on both xenon samples.

In Figure 3A, the maximum achievable polarization of the sample doped with 30 mM TEMPO increased from 7.8% ± 0.8% to 22.6% ± 2.4%, and the buildup time constant at the frequency providing the best enhancement decreased from 7292 ± 700 s to 3742 ± 350 s (see the Supporting Information for buildup times and spectra). The latter was a clear indication of the fact that microwave frequency modulation triggered longer range spectral diffusion, allowing the use of a broader portion of the ESR spectrum and yielding a faster and more effective polarization transfer to the ^129^Xe nuclei. In Figure 3B, for the xenon sample doped with 50 mM TEMPO, the effect of microwave frequency modulation was less pronounced, because of the higher radical concentration. Nevertheless, the maximum achievable polarization increased from 15.3% ± 1.6% to 24.3% ± 2.2%, and the buildup time constant, at the frequencies providing the best enhancement, decreased from 2400 ± 250 s to 1925 ± 200 s (see the Supporting Information). In Figure 3D, we summarize the DNP performance improvement for the three samples: applying microwave frequency modulation increased the DNP enhancement by 189%, 59%, and 5% for the xenon sample with 30 mM TEMPO, the xenon sample with 50 mM TEMPO, and the acetate sample, respectively. Note that, despite a slower buildup time constant, applying microwave frequency modulation equalized the maximum achievable polarization of the two xenon samples, providing a sort of empirical limit at the given radical type and experimental conditions. More importantly, the polarization of the xenon samples exceeded that of the carbon sample upon employment of microwave frequency modulation. Given that the ratio between the ^129^Xe and ^13^C gamma factors was smaller than the ratio between the maximum polarizations achieved by the two nuclei, some other factors must have come into play. Surprisingly, the addition of xenon to the radical-doped isobutanol not only decreased the *T*_1e_, but also reduced the inhomogeneous line width of the radical spectrum (see the Supporting Information). It is well-known that narrower line radical such as trityl are beneficial for low-gamma nuclei polarization,^54^ and that xenon affects the g-tensor of paramagnetic molecules in the surroundings.^45^ Although our observation deserves further investigation, it is consistent with the Borghini model, where a reduction of the “thermal heat capacity” of the non-Zeeman reservoir (i.e., a reduction of the breadth of the total ESR line) would predict a higher DNP enhancement.^55−57^

In the present study, we investigated xenon DNP at 5 T and 1.20 K in detail and the reasons why it generally underperforms with respect to ^13^C DNP, despite the two gyromagnetic ratios being close in value. Our findings revealed that the main cause was the effect on the radical spin–lattice relaxation time (*T*_1e_) of the low-melting-point glassing solvent (i.e., isobutanol) used during sample preparation, which allows mixing with xenon in proximity of its triple point’s temperature. Although this study focused on isobutanol, a similar behavior was also observed for other alcohols (e.g., ethanol and 2-methyl-1-pentanol). Differently, ordinarily used glassing mixtures for the preparation of ^13^C DNP samples (e.g., glycerol:water) create a radical environment such that the *T*_1e_ is 5 times longer with respect to xenon matrices, at least. We suggested using microwave frequency modulation as the best option to circumvent this drawback and achieve over 20% xenon polarization at 5 T and 1.20 K without employing any expensive deuterated solvent. Also, thanks to the CFP technology combined with ultrasonication at controlled temperature, we improved the preparation method and the quality/homogeneity of the samples.

Besides the pure physicochemical interest of this study, developing further xenon dDNP methods could open new perspectives, in terms of HP MR applications. For instance, brain perfusion and brain metabolism can be investigated by employing HP xenon and HP [1-^13^C]pyruvate, respectively. Currently, one would need a SEOP polarizer and a dDNP polarizer to run both experiments in the same subject.^58,59^ Pushing xenon DNP methods further by improving polarization level and augmenting the gas volume produced, combined with a dDNP polarizer with multisample capability,^60^ would allow the use of the same machine for both purposes, saving financial resources and reducing the instrumentation footprint.

## Materials and Methods

### Sample Preparation

In this work, we prepared three kinds of samples: ^129^Xe DNP samples, ^13^C DNP samples, and LOD only samples. All chemicals in liquid or powder form at room temperature were purchased from Sigma–Aldrich (Buchs, Switzerland). Xenon gas was purchased from Messer AG (Lenzburg, Switzerland).

#### ^129^Xe DNP Samples

**Figure 4:**
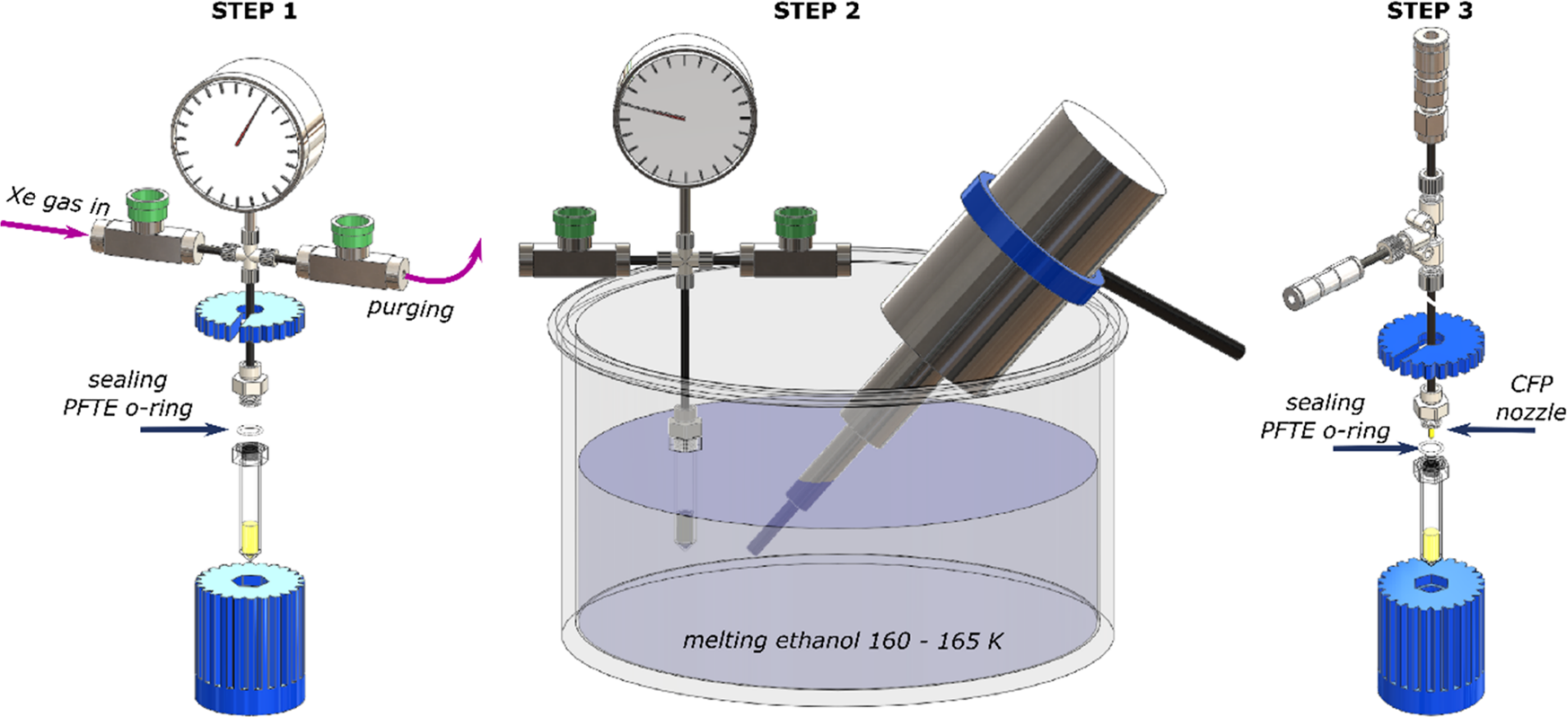
Sketch of the procedure/setup used to prepare xenon DNP samples at controlled temperature conditions and directly inside the CFP’s vial. **Step 1**: the vial containing the radical-doped glassing solvent is sealed to the custom-made xenon gas dispenser using purposely made 3D printed wrenches; then, the volume between the inlet and outlet is purged with xenon gas; finally, a defined amount of xenon gas (pressure-gauge-regulated) is introduced in the system, the inlet and outlet are closed, and the xenon gas bottle (not shown) is disconnected. **Step 2**: the vial is introduced in a bath of melting ethanol (160–165 K) together with an ultrasound probe (20 kHz, 500 W); the gas is slowly condensed on top of the radical doped solvent and dissolved into it by means of ultrasonication. **Step 3**: the xenon gas dispenser is lifted out from melting ethanol and quickly frozen in liquid nitrogen; the vial, continuously kept in liquid nitrogen for half of its height, is disconnected from the xenon gas dispenser, sealed to the CFP using a new PTFE O-ring and tested for leaks as earlier described, prior to being loaded inside the polarizer.^23,61^

Differently from previous works,^13,29^ we herein employ the Custom Fluid Path (CFP) technology^27,61^ to load samples inside the polarizer. Therefore, ^129^Xe DNP samples were directly prepared inside a purposely made 450 μL Kel-F see-through vial (Figure 4).

For all xenon samples in this study, we chose to work with only one solvent, and one xenon concentration (i.e., 2.6 M) well below the solubility threshold previously found for isobutanol,^29^ to avoid spin diffusion effects between the dissolvent xenon compartment and the pure xenon compartment, and therefore simplify the interpretation of the observed phenomena. The sample preparation procedure was performed in three steps. In **step 1**, 200 μL of TEMPO radical-doped solvent were pipetted inside the vial. The latter was sealed, using a polytetrafluoroethylene (PTFE) O-ring, to a custom-made gas dispenser, and the volume above the liquid was carefully flushed with xenon gas (4.0 purity) supplied by a 1 L bottle at 12 bar (not shown in the figure). The total volume of vial + dispenser was 2.2 mL, requiring a xenon gas pressure of 6.5 bar to obtain the desired concentration of xenon in the solvent and a consequent volume increase of the solution of ∼10%. Thus, to make xenon samples with TEMPO concentrations of 30 and 50 mM, two stock solutions of isobutanol and TEMPO radical at 34 and 56 mM, respectively, were initially prepared. In **step 2**, after disconnecting the dispenser from the Xe bottle, the vial was plunged in a bath of melting ethanol at a temperature of 160–165 K to slowly condense the gas on top of the solvent and mix both phases together by means of a 20 kHz/500 W ultrasound probe (Model VC505 ultrasonic processor, Sonics & Materials, Newtown, CT, USA). This procedure lasted for ∼5 min until the pressure gauge dropped to zero, meaning that all the xenon was incorporated inside the solvent. In **step 3**, the vial was quickly removed from the ethanol bath, wiped, and plunged in liquid nitrogen to flash-freeze the sample. Finally, the vial was disconnected from the dispenser, sealed to the CFP using a new PTFE O-ring and prepared for the insertion into the DNP polarizer as previously described.^60,61^

As a comparison, a sample containing 50 mM TEMPO was prepared as previously described,^29^ placing in a custom-designed glass cold finger 500 μL of radical-doped glassing solvent and xenon at a defined pressure to obtain a final concentration of 2.6 M. Condensation of the gas and mixing with the solvent were achieved by cooling the system in liquid nitrogen, freezing both compounds, warming them up until both were in the liquid phase, quickly stirring them using a bar magnet placed inside the cold finger, and cooling again the solution below the freezing point to limit sublimation of the gas. This procedure was repeated a certain number of times until the pressure gauge was showing no residual gas pressure, prior to transferring part of the sample into the CFP vial.

The sample homogeneity obtained using the original and new preparation procedures was evaluated from the Free Induction Decay (FID) of the NMR signal.

#### ^13^C DNP Samples

We also prepared a typical carbon DNP sample containing 3 M sodium [1-^13^C]acetate dissolved in glycerol:water 50:50 (v/v) (hereafter referred to as GW55) doped with 30 mM 4-hydroxy-TEMPO. For this preparation, we did not use the setup described above. We simply mixed into a microcentrifuge tube sodium [1-^13^C]acetate and glycerol:water in ratios to obtain a final concentration of 3 M for the salt. The solution was sonicated for 10 min at 40 °C to ensure complete dissolution of the acetate. One milliliter (1 mL) of this stock solution was then pipetted into a second microcentrifuge tube, doped with an amount of 4-hydroxy-TEMPO to obtain a final concentration of 30 mM for the radical, and sonicated at 40 °C for another 5 min. Finally, 200 μL of the liquid sample were pipetted inside the vial, sealed to the CFP, leak-tested and prepared for DNP as previously described.^60,61^

#### LOD Samples

LOD-ESR measurements were performed on the samples described above, as well as on frozen solutions of isobutanol doped with 30 mM/50 mM TEMPO and GW55 doped with 30 mM 4-hydroxy-TEMPO. This investigation had the aim of assessing the influence of the presence of the substrate (i.e., xenon or sodium [1-^13^C]acetate) on the radial properties.

### DNP Measurements

DNP was performed on all samples using a polarizer working at 5 T and 1.150 ± 0.05 K. The polarizer (Vanderklink Sarl, La Tour-de-Peilz, Switzerland), built around a “wet” cryostat, is similar, with regard to working principle and design of the prototype developed by Comment et al. in 2007,^62^ and more recently upgraded by Lê et al. in 2022 with a lower He consumption/higher performance DNP probe, the CFP technology, and a Cameleon 4 Gecho spectrometer (RS2D, Mundolsheim, France) for monitoring the NMR signal. To measure both ^129^Xe and ^13^C, the pseudo-Alderman-Grant coil inside the microwave cavity was connected to two different remote tuning/matching networks and passive T/R switches resonating at 53.44 and 58.78 MHz, respectively.

First, for each sample, a microwave frequency sweep was performed from 139.6 to 140.5 GHz, in steps of 40 MHz, with and without microwave frequency modulation (i.e., 1 kHz of modulation rate, 100 MHz modulation amplitude), for measuring the DNP spectrum of the sample and determining optimal irradiation conditions (*n* = 1). For each frequency step, the sample was hyperpolarized using a microwave power of 55 mW, and the NMR signal was acquired with a 30° hard pulse after 10 min. Before each polarization interval, microwaves were switched off and the polarization destroyed with 1000× 10° hard pulses.

Second, at optimal DNP conditions, the sample was hyperpolarized and monitored with a 5° hard pulse every 120 s to measure the buildup curves (*n* = 2). Depending on the sample, the experiment lasted 2–4 h. A monoexponential curve was fit to all polarization buildup data to estimate the buildup time constant *T*_b_.

Finally, the maximum achievable HP signal was read out with a 18° hard pulse to obtain higher SNR. The solid-state DNP enhancement factor (ε) was computed by dividing the spectrum’s integral of the above-mentioned signal by the one measured, at the same NMR parameters, after complete relaxation to thermal equilibrium (*n* = 2). The corresponding solid-state polarization was inferred by multiplying ε by the Boltzmann value at 5 T and 1.20 K.

### LOD Measurements

As previously described,^61^ the LOD-ESR probe, mounted using the outer lumen and dynamic seal of a separate fluid path, could be slid inside the DNP probe, without modifying any critical part of the hardware and perform the ESR investigation at same DNP conditions of microwave, field, and temperature. For electron *T*_1_ (*T*_1e_) measurements, the microwave output power was modulated at 1 Hz for samples with isobutyl alcohol or 0.33 Hz for samples with GW55 between 0 and 35 mW (maximum available output power after mounting a voltage-controlled attenuator at the output of the source). The rate was low enough to record the complete evolution of the electron spins’ signal as a function of time during saturation and relaxation; the signal was averaged 30–100 times, depending on the single acquisition’s SNR. Extraction of the *T*_1e_ was performed by fitting the equation *S*(*t*) =  to the time-course data of the signal *S*: here, *A* is a free parameter that represents the highest signal intensity, and τ is the characteristic time constant of the LOD-ESR probe (i.e., 20 ms), measured by exciting the setup with a squared wave.^23^ For radical spectrum recording, the microwave frequency was increased from 139.6 to 140.5 GHz in steps of 5 MHz and the output power modulated at 4.8 Hz. For each frequency step, the demodulated signal was integrated for 10 s in the time domain, equivalent to set the low pass filter of the lock-in amplifier of the homemade LOD-ESR spectrometer to 0.1 Hz.

### Data Processing and Statistical Analysis

All LOD-ESR data, acquired in LabView 2019 (National Instruments, Austin, TX, USA) were processed in MATLAB 2023 (The MathWorks, Natick, MA, USA). DNP/NMR data acquired in SPINit 2020.06 (RS2D, Mundolsheim, France) were exported as JCAMP-DX files, processed in MNova (MestreLab Research, Santiago, Spain), and then in MATLAB 2023. All polarizations and relaxation/saturation time constants are presented as the mean ± standard deviation of repeated measurements.

## Supporting Information Available

*T*_1e_ raw data for all samples; buildup time curves at optimal microwave irradiation; comparison between ESR spectra of TEMPO in isobutanol with and without xenon; NMR spectra of thermal equilibrium and DNP enhanced signals. Transparent Peer Review report available (PDF)
Video 1: xenon sample preparation employing ultra sonication at low temperature (MP4)
