# Supplementary material for: 129Xe Dynamic Nuclear Polarization Demystified: The Influence of the Glassing Matrix on the Radical Properties
Source: J Phys Chem Lett. 2024 Mar 7;15(11):2957–65. doi: 10.1021/acs.jpclett.4c00177 (PMC10961830; doi:10.1021/acs.jpclett.4c00177)
Supplement: Supplementary file 1 — jz4c00177_si_001.pdf [file jz4c00177_si_001.pdf]

Supporting Information

^{129}Xe Dynamic Nuclear Polarization Demystified: the Influence of the Glassing Matrix on the Radical Properties.

Emma Wiström,[1] Jean-Noël Hyacinthe,[1] Thanh Phong Lê,[1] Rolf Gruetter,[1] and Andrea Capozzi.[1,2,]*

[1]LIFMET, Institute of Physics, École Polytechnique Fédérale de Lausanne (EPFL), Station 6, 1015 Lausanne (Switzerland).

[2]HYPERMAG, Department of Health Technology, Technical University of Denmark, Building 349, 2800 Kgs Lyngby (Denmark).

Corresponding Author

*Dr. Andrea Capozzi

EPFL SB IPHYS LIFMET, CH F1 633 (Bâtiment CH), Station 6

CH-1015 Lausanne

Email: andrea.capozzi@epfl.ch; Tel: +41 21 693 05 88;

ORCID: 0000-0002-2306-9049

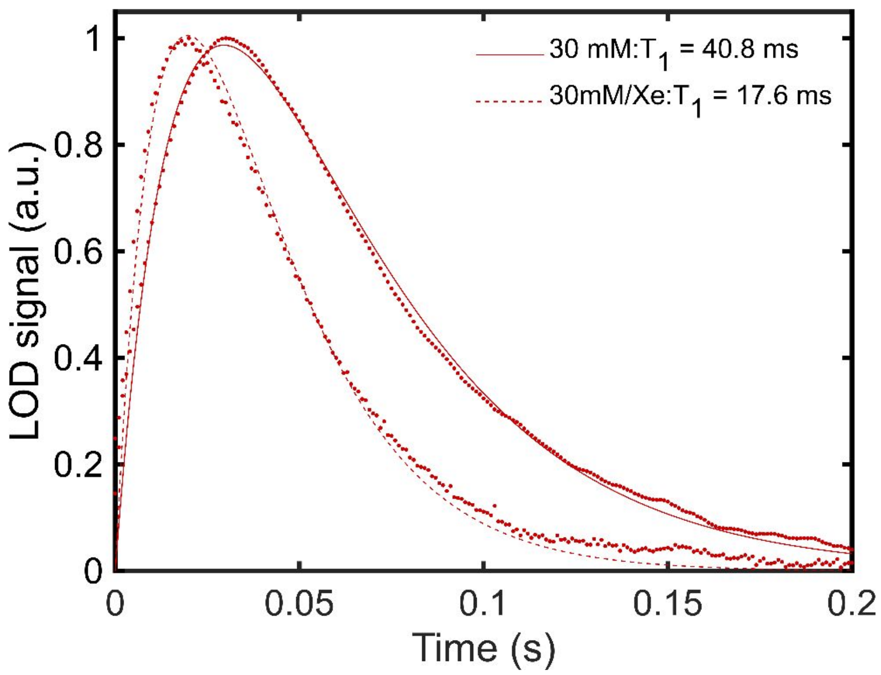

Figure S1. Radical spin-lattice relaxation for 30 mM TEMPO in isobutanol with and without 2.6 M xenon. The time constants are reported on the graph.

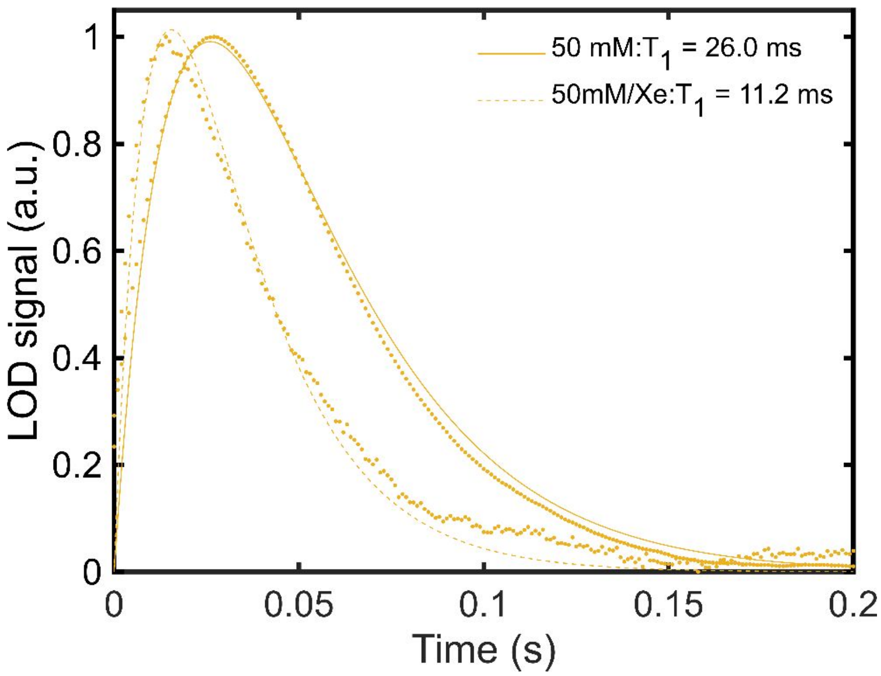

Figure S2. Radical spin-lattice relaxation for 50 mM TEMPO in isobutanol with and without 2.6 M xenon. The time constants are reported on the graph.

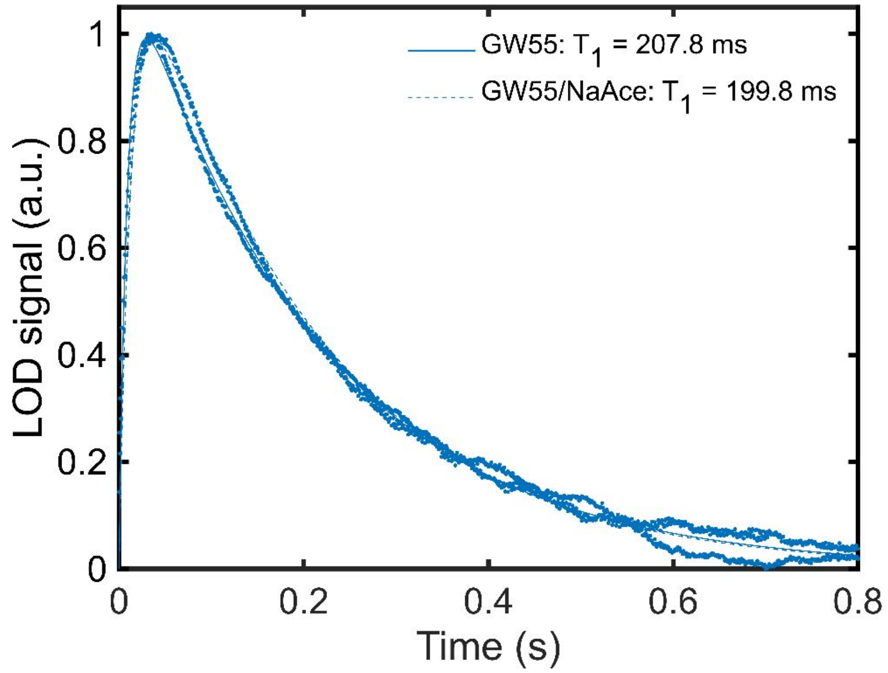

Figure S3. Radical spin-lattice relaxation for 30 mM TEMPOL in GW55 with and without 3M sodium [1-^{13}C]acetate. The time constants are reported on the graph.

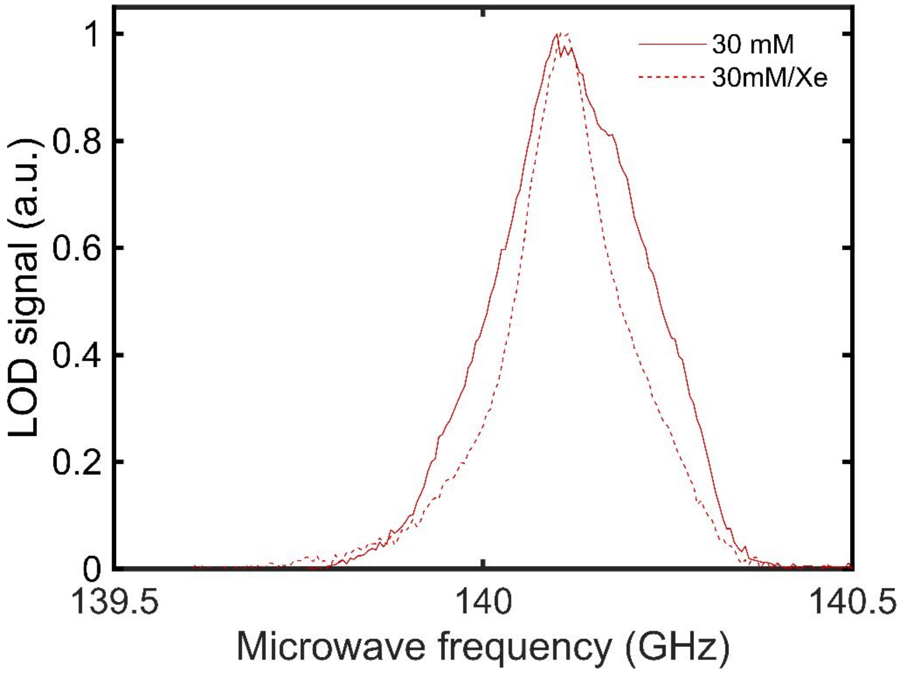

Figure S4. LOD-ESR spectra of 30 mM TEMPO in isobutanol with and without 2.6 M xenon.

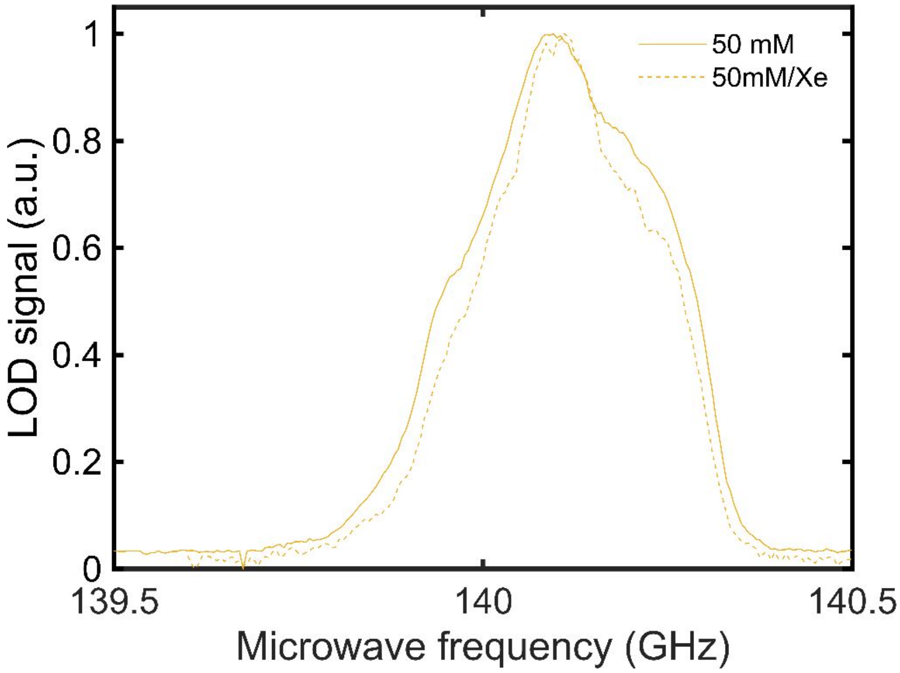

Figure S5. LOD-ESR spectra of 50 mM TEMPO in isobutanol with and without 2.6 M xenon

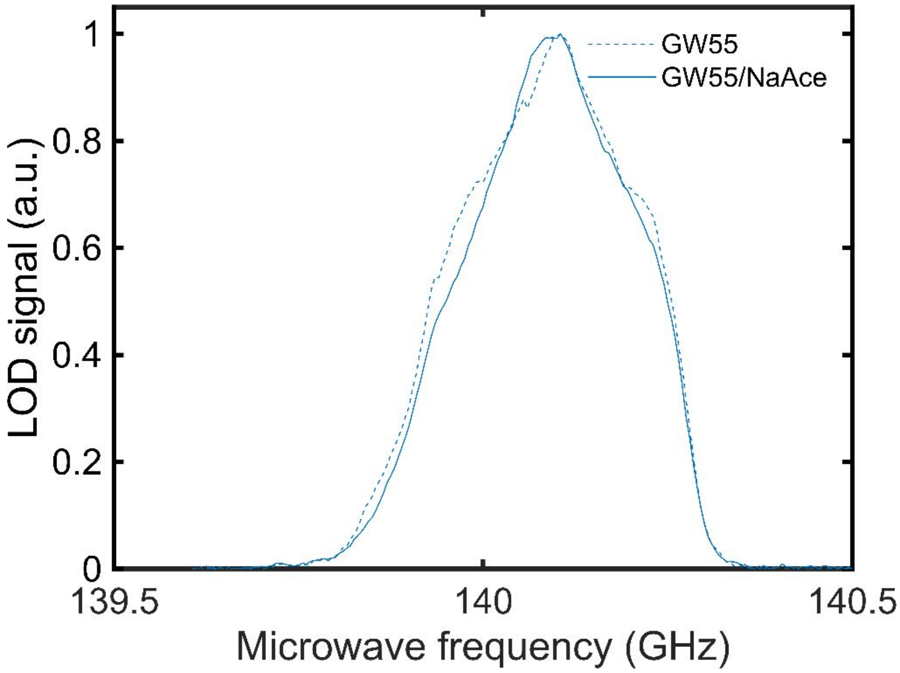

Figure S6. LOD-ESR spectra of 30 mM TEMPOL in GW55 with and without 3M sodium [1-^{13}C]acetate.

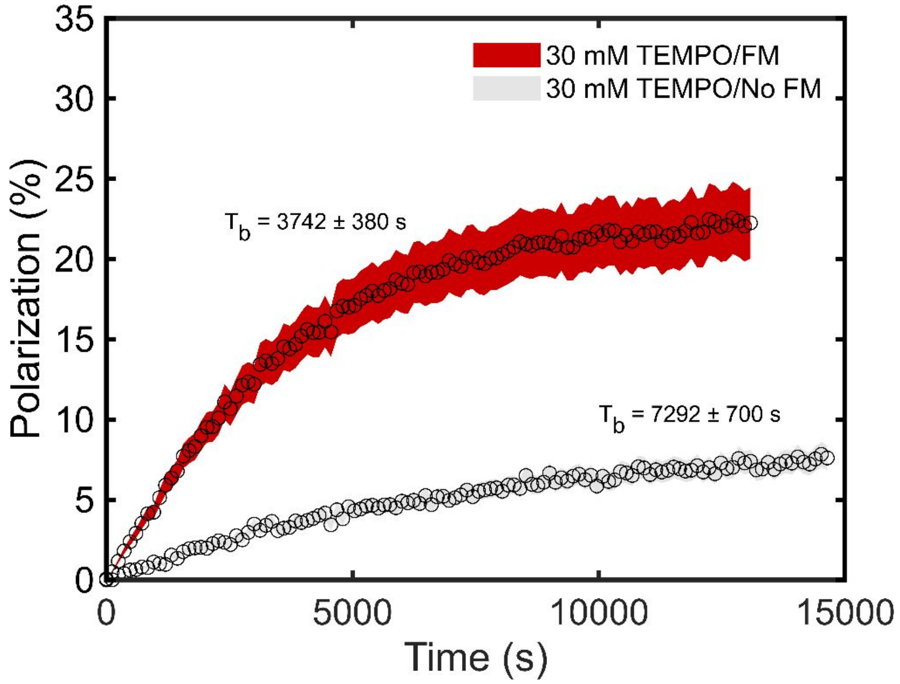

Figure S7. Polarization buildup curves for 30 mM TEMPO in isobutanol and 2.6 M xenon, with and without microwave frequency modulation at best irradiation conditions. Mono-exponential buildup time constants are reported on the graph.

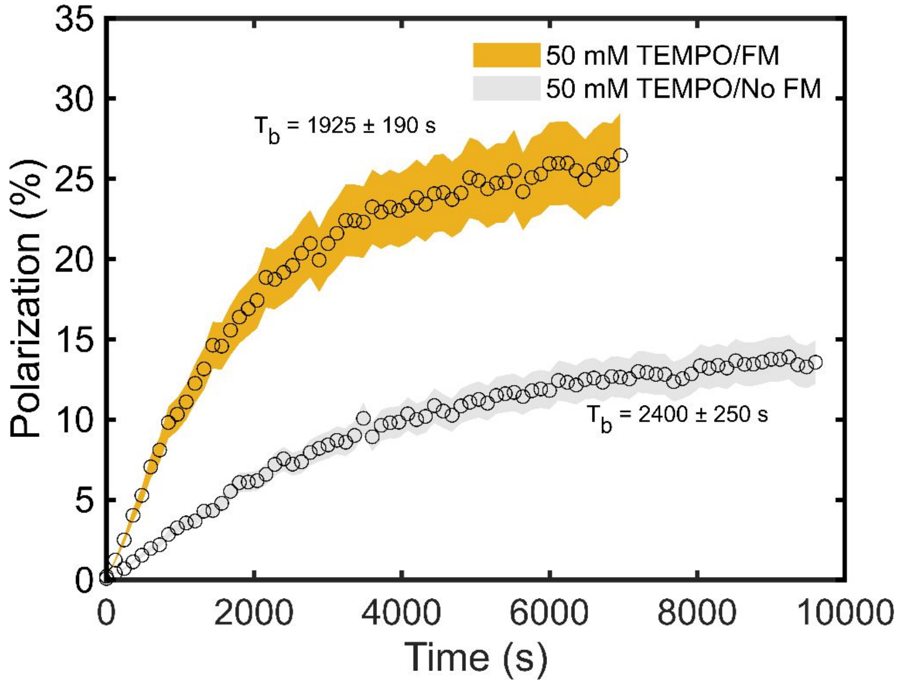

Figure S8. Polarization buildup curves for 50 mM TEMPO in isobutanol and 2.6 M xenon, with and without microwave frequency modulation at best irradiation conditions. Mono-exponential buildup time constants are reported on the graph.

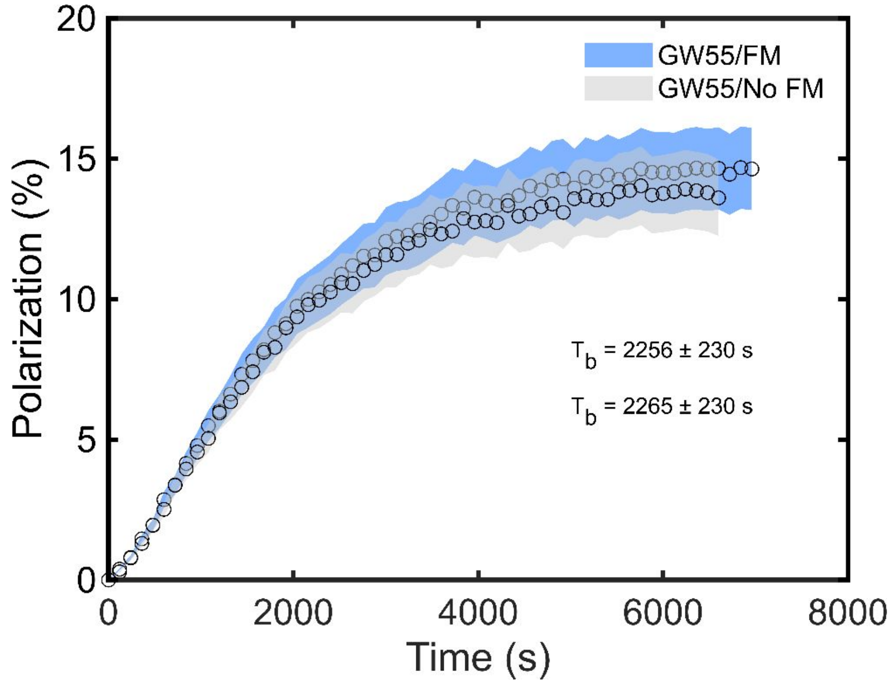

Figure S9. Polarization buildup curves for of 30 mM TEMPOL in GW55 and 3M sodium [1-^{13}C]acetate, with and without microwave frequency modulation at best irradiation conditions. Mono-exponential buildup time constants are reported on the graph.

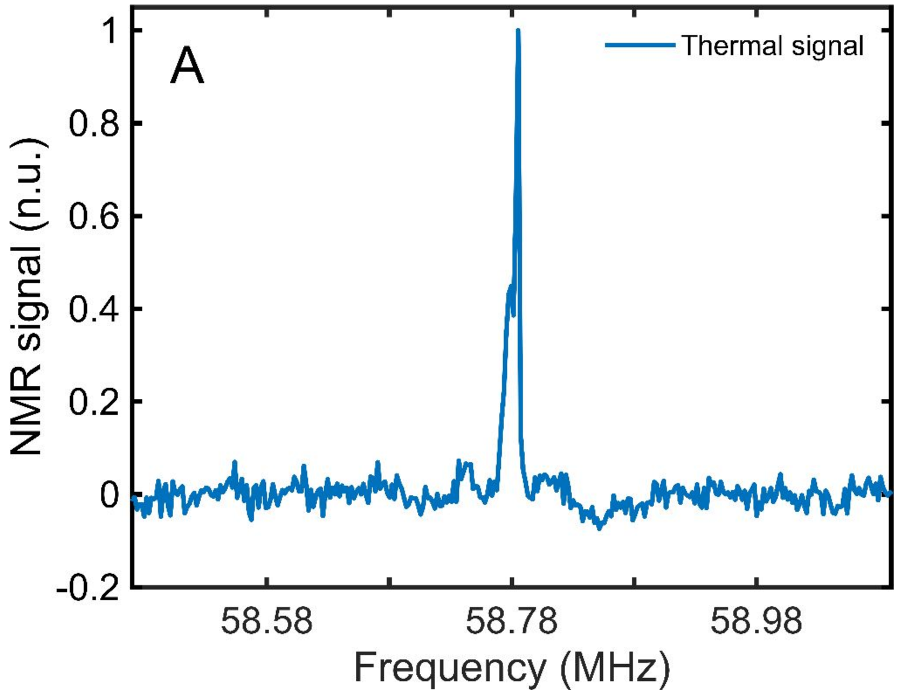

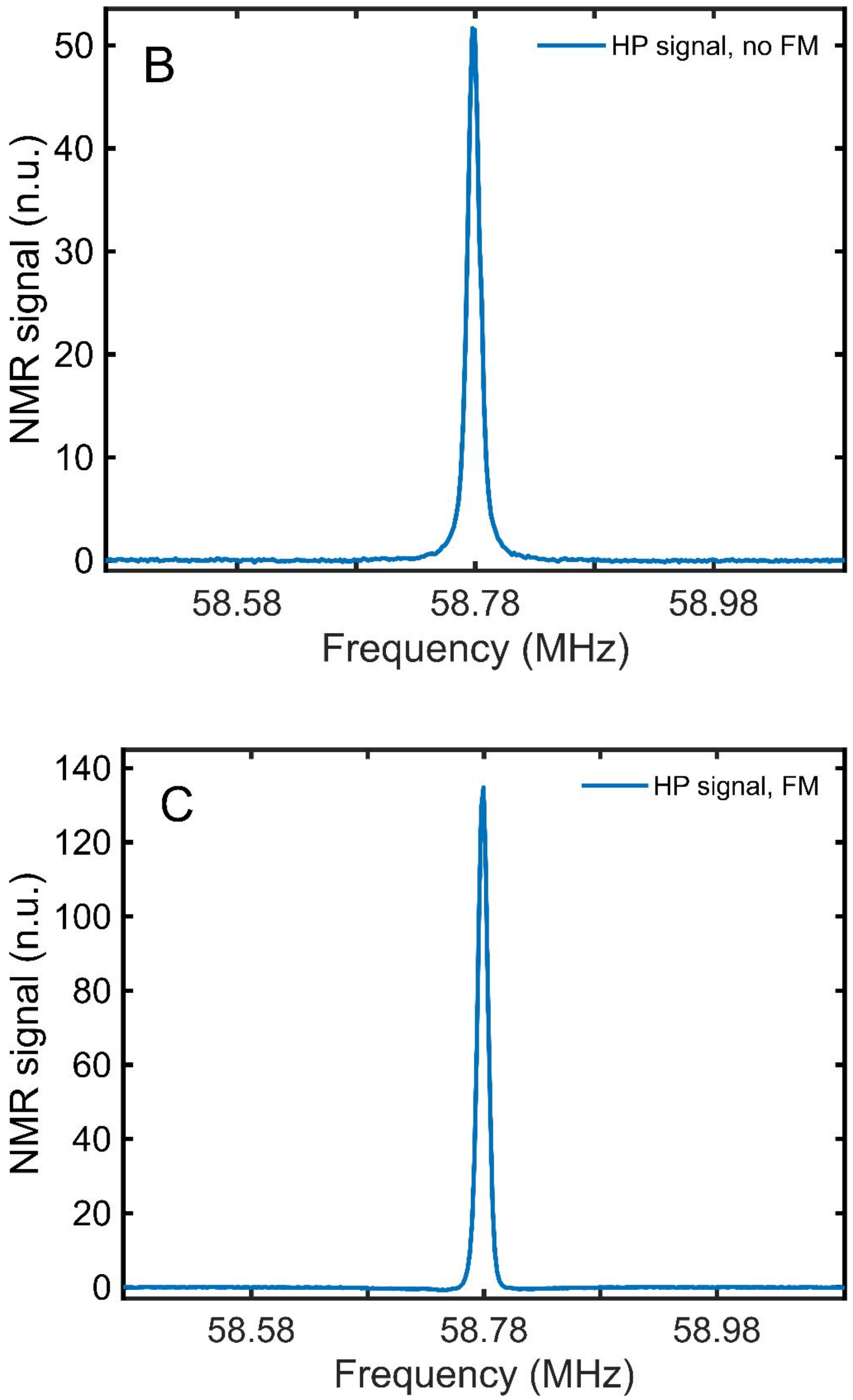

Figure S10. NMR spectrum of 30 mM TEMPO in isobutanol and 2.6 M xenon measured after complete relaxation/polarization. Signal at thermal equilibrium (**A**); hyperpolarized signal without microwave frequency modulation (**B**); hyperpolarized signal with microwave frequency modulation (**C**). All processed data have zero line broadening and are normalized to the amplitude of the thermal equilibrium signal.
