# Supplementary material for: 129Xe Dynamic Nuclear Polarization Demystified: The Influence of the Glassing Matrix on the Radical Properties
Source: J Phys Chem Lett. 2024 Mar 7;15(11):2957–65. doi: 10.1021/acs.jpclett.4c00177 (PMC10961830; doi:10.1021/acs.jpclett.4c00177)
Supplement: Supplementary file 3 — jz4c00177_si_003.pdf [file jz4c00177_si_003.pdf]

jz-2024-00177m.R1

Name: Peer Review Information for "¹²⁹Xe Dynamic Nuclear Polarization Demystified: The Influence of the Glassing Matrix on the Radical Properties"

First Round of Reviewer Comments

Reviewer: 1

Comments to the Author

1. What is the major advance reported in the paper?

The paper focus on hyperpolarization of 129Xe with dDNP method, rather than the SEOP method. And introduced two solutions for preparing sample and increasing polarization.

These could be helpful for developing dDNP method for the gas similier to Xe, like krypton. Regard to hyperpolarization method itself, the paper also give some perspective about the ESR's influence on Xenon's polarization, which could be a source of reference.

2. What is the immediate significance of this advance?

A method user-friendly method for Xeon sample preparation, and the typical ESR method to increase the polarization of Xenon.

3. Technical suggestions

As the authors emphasized in the draft, the dDNP method for hyperpolarization of 129Xe seem not suitable for applications, such as clinic. However, it's still worth to try dDNP method to reveal something that SEOP can not.

1. Develope the dDNP method for Noble gas

2. Find the advantages of dDNP for Xenon polarization

Reviewer: 2

Comments to the Author

The manuscript presents a comprehensive comparison of two hyperpolarization techniques for 129Xe: Dynamic Nuclear Polarization (DNP) and Spin Exchange Optical Pumping (SEOP).

The study is inspired by an intriguing question: why is the performance of 129Xe DNP so different from that of 13C, despite the close gyromagnetic ratios of the two?

A Custom Fluid Path (CFP) system was introduced for consistent and effective sample preparation of 129Xe. This system enabled homogeneous xenon DNP samples by meticulously managing temperature conditions.

By developing this new sample preparation method and employing microwave frequency modulation, the research overcame previous challenges in129Xe DNP, achieving over 20% polarization without the need for deuterated solvents.

Additionally, the research explored the effects of different glassing matrices on radical properties, discovering that certain matrices significantly shorten the electron T1 relaxation times.

The use of microwave frequency modulation proved beneficial for improving DNP in such scenarios, allowing higher 129Xe polarization levels without the necessity for deuterated solvents.

Overall, I find the paper to be a valuable contribution to the field and recommend its publication, subject to the clarification of a few key points.

A reason for the low DNP efficiency in 129Xe could also be inefficient spin diffusion, as it has a larger chemical shift dispersion compared to 13C. I suggest that the authors delve deeper into this topic. Exploring this aspect could provide a clearer understanding of the limitations associated with Xenon DNP.

Can the authors elucidate the plausible underlying mechanisms for the faster relaxation in the Xenon glassing matrix?

The paper mentions that Xe-SEOP exhibits higher throughput compared to d-DNP. It would be beneficial for the authors to elaborate on the circumstances under which d-DNP would offer advantages over Xe-SEOP, especially given the potentially higher cost of d-DNP.

The inclusion of an NMR spectrum of hyperpolarized Xenon would be highly beneficial. This would provide empirical evidence to support the findings.

Author's Response to Peer Review Comments:

Dear Editor,

Thanks for your message. We are very happy about the overall positive outcome of our first submission. This is a subject that required some years of study, and we are very excited about the fact that JPCL is interested in publishing a revised version of the paper. We did our best to fulfil the reviewers' requirements, as well as yours, needed to improve the manuscript. Below, you can find, in the order, the list of all comments (in black) and our point-by-point responses (in red). For each reviewer we also added a general answer at the beginning of the concerned paragraph of this document. Upon re-submission, we uploaded two versions of the main manuscript: one with all changes highlighted in yellow, and a clean one. Also a revised version of SI was resubmitted.

Answers to editor's comments:

2. Title: In both the main manuscript file and the Supporting Information, set the title in title case, with the first letter of each principal word capitalized.

OK

3. Abstract: Shorten the abstract to 150 words or fewer.

OK

4. TOC Graphic: Please resize the TOC graphic per journal guidelines (2 in x 2 in).

OK

5. Headers: Remove the section heading(s) throughout the body of the manuscript (you can leave Methods and Abstract headings).

OK

6. References: In both the main file and the supporting information, fix the style of all references to use

JPCL formatting (check all references carefully). ***JPC Letters reference formatting requires that journal references should contain: () around numbers, author names, article title (titles entirely in title case or entirely in lower case), abbreviated journal title (italicized), year (bolded), volume (italicized), and pages (first-last). Book references should contain author names, book title (in the same pattern), publisher, city, and year. Websites must include date of access.
OK, all DOIs were removed after page numbers. Ref 4 that is online medical newspaper with no pages and authors specified, was modified as follows:
(4) FDA Approves Hyperpolarized Xenon for MRI. AppliedRadiology®. Website:

https://appliedradiology.com/articles/fda-approves-hyperpolarized-xenon-for-mri. Date of access: Feb

12th, 2024.

7. Supporting Information: Please number SI pages in the following format: “S1, S2...”

OK

8. Graphics: One or more of your figure legends includes a citation. Permission is required if you are using another publisher's or copyright owner's figures/tables verbatim, adapting or modifying them, or using them in part. Permission may not be required if you are only using data to create your figure. If this is the case, please notify our office. Additionally, permission is not required when images are reused from ACS publications.

All references in the figures’ legends do not refer to any third-party image. All figures in the main manuscript and SI were genuinely produced by the authors of the manuscript and never published before. References 23, 29, 53 reported in the legend of Fig.1 and Fig.4 are related to sample preparation/loading procedures explained in previous works but have nothing to do with the figures reported in these manuscripts.

Answers to Reviewer 1

Recommendation: This paper may be publishable, but major revision is needed; I would like to be invited to review any future revision.

Comments:

As the authors emphasized in the draft, the dDNP method for hyperpolarization of 129Xe seem not suitable for applications, such as clinic. However, it’s still worth to try dDNP method to reveal something that SEOP cannot.

1. Develop the dDNP method for Noble gas

2. Find the advantages of dDNP for Xenon polarization

We thank the reviewer for his/her overall positive feedback and the acknowledged possible interest that the physical chemistry community can show with respect to our work. Coming to his/her comments, we would like to stress the fact that, as already explained in the introduction, as of today, Xenon dDNP stands no chances with respect to SEOP when it comes to clinical applications. SEOP is faster, produces higher volumes and, most importantly, this technology was pushed to the point of producing “ready to breath by humans” hyperpolarized gas. The main aim of this work is a genuine physical chemistry study with no applications in mind. That has been said, and as already mentioned in the introduction, what dDNP has to offer is versatility. Since all NMR active nuclei can in theory be polarized, one could think about performing back-to-back injections of different compounds to investigate different features of the same pathology. For instance, we could think about monitoring the brain perfusion using HP Xenon (https://doi.org/10.1148/radiol.2017162881) and the brain metabolism using HP carbon (10.1016/j.neuroimage.2019.01.027). Currently, this kind of study would require a SEOP polarizer for xenon and a dDNP polarizer for carbon. Differently, a further improvement of Xenon DNP plus the employment of a multi-sample dDNP polarizer could allow to use the same machine for both contrast agents. Finally, the development of dDNP methods for other noble gases is beyond the scope of this study.
To fulfil the reviewer’s requirement, we added the following perspective paragraph in the conclusion:

“Besides the pure physicochemical interest of this study, developing further xenon dDNP methods could open new perspectives in terms of HP MR applications. For instance, brain perfusion and brain metabolism can be investigated employing HP xenon and HP [1-13C]pyruvate, respectively. Currently, you would need a SEOP polarizer and a dDNP polarizer to run both experiments in the same subject.56,57 Pushing xenon DNP methods further by improving polarization level and gas’s volume produced, combined with a dDNP polarizer with multi-sample capability,58 would allow to

use the same machine for both purposes, saving financial resources and reducing the instrumentation footprint."

Answer to Reviewer 2

Overall, I find the paper to be a valuable contribution to the field and recommend its publication, subject to the clarification of a few key points.

We thank the reviewer for the overall appreciation of our work and his/her recommendation for publication of a revived version of the manuscript.

A reason for the low DNP efficiency in 129Xe could also be inefficient spin diffusion, as it has a larger chemical shift dispersion compared to 13C. I suggest that the authors delve deeper into this topic. Exploring this aspect could provide a clearer understanding of the limitations associated with Xenon DNP.

The reviewer's suggestion is very interesting and drew all our attention. It is correct that the chemical shift dispersion of xenon is larger than carbon, but as far as the xenon is not in two different compartments, this is not a problem for solid-state DNP. We investigated this issue in a previous work published in 2015 in JPCC (https://doi.org/10.1021/jp5124053). There, we studied the influence of xenon concentration in the sample on the polarization level/DNP mechanism. We found that when the xenon is beyond the solubility threshold for a given solvent, the DNP spectrum appears as a doublet: a broad peak (xenon dissolved in the glassing agent and doing direct DNP) and a thin peak (pure xenon crystals that are too far from the radical – dissolved in the glassing agent – and polarized via spin diffusion between the two compartments). In Figure 2 of that work, you can have a clear picture of the situation.

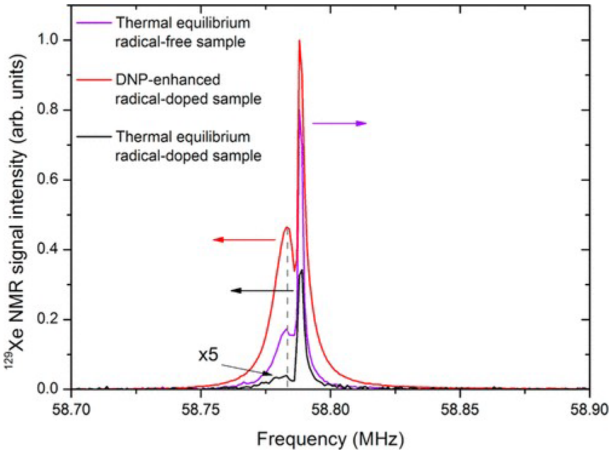

Differently, if you prepare samples with a xenon concentration below the solubility threshold, you still see a different chemical shift depending on the kind of solvent, but all the xenon is polarized directly by the radical (Figure 3 of that work).

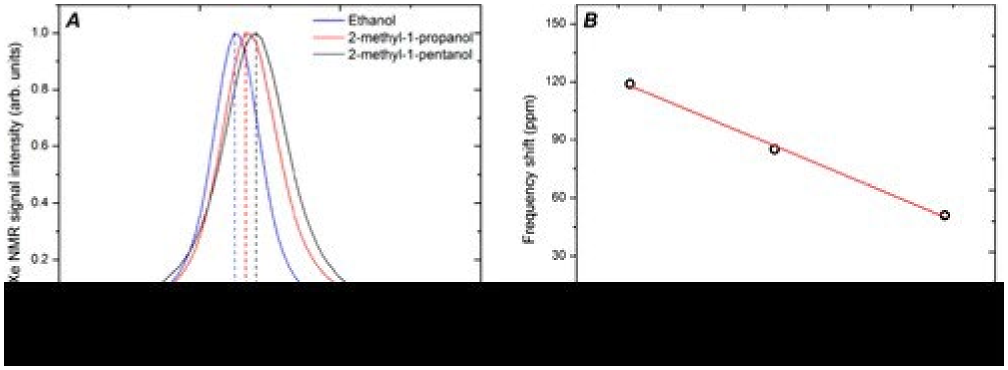

[redacted]

Indeed, for a given solvent, the maximum achievable polarization achieves a plateau for Xenon concentrations ≤ of the solubility threshold (Figure 1 of that work, the small arrow indicated the solubility threshold for a given solvent).

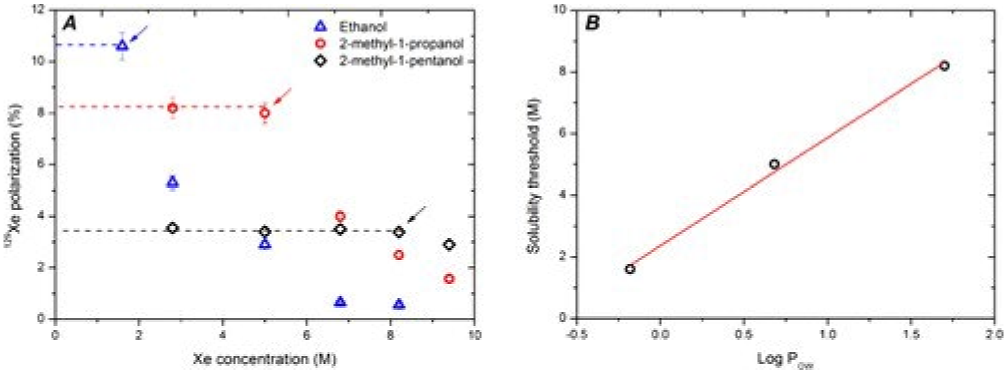

More concentrated xenon solvent (but below solubility threshold) will achieve the polarization plateau faster because of stronger spin diffusion, but this will not change the maximum achievable polarization.
As stated in the methods, in this study we deliberately chose to work “below solubility threshold” conditions for isobutanol to push nuclear spin diffusion out of the game. To clarify this important point we added the following sentence in the methods.
“For all xenon samples in this study, we chose to work with only one solvent, and one xenon concentration (i.e. 2.6 M) well below the solubility threshold, previously found for isobutanol,29 to avoid spin diffusion effects between the dissolvent xenon compartment and the pure xenon compartment and, therefore, simplify the interpretation of the observed phenomena”.

Can the authors elucidate the plausible underlying mechanisms for the faster relaxation in the Xenon glassing matrix?
Thanks for stressing this point. We provided the following explanation in the results and discussion session

“Although the precise underlying mechanisms for the faster electron spins relaxation in xenon samples is still unclear and would require further investigation, we can for sure consider that,

differently from nuclear spins, the most effective relaxation mechanism for electron spins in the solid state is the coupling with lattice vibrations (i.e. phonon modes).42 The phonons in a solid, be it a crystal or a glass, are a basic ingredient in understanding such things as specific heat, melting, ferroelectricity and superconductivity.43 The melting temperature of isobutanol (i.e. 165 K) is much lower compared to the one of glycerol:water at equal volume percentage (i.e. 245 K)44. Therefore, the phonons spectral density of the two glassing matrices is likely to be very different, with the intensity of the phonon spectral density around 140 GHz being stronger for isobutanol than for glycerol:water. Within the same reasoning, further shortening of the T1e upon admixture of xenon in the matrix is coherent with the fact that the gas melting point falls at 161 K."

The paper mentions that Xe-SEOP exhibits higher throughput compared to d-DNP. It would be beneficial for the authors to elaborate on the circumstances under which d-DNP would offer advantages over XeSEOP, especially given the potentially higher cost of d-DNP.

See answer to reviewer 1

The inclusion of an NMR spectrum of hyperpolarized Xenon would be highly beneficial. This would provide empirical evidence to support the findings.
We added NMR spectra of thermally polarized Xenon, DNP polarized Xe without microwave modulation and DNP polarized Xenon with microwave frequency modulation in Supporting Information and added the following sentence in the results and discussion session of the main text.
"(see Supporting Information for buildup times and spectra)".
